# Early repolarization with horizontal ST segment may be associated with aborted sudden cardiac arrest: a retrospective case control study

**DOI:** 10.1186/1471-2261-12-122

**Published:** 2012-12-11

**Authors:** Sung Hea Kim, Do Young Kim, Hyun-Joong Kim, Sang Man Jung, Seong Woo Han, Soon Yong Suh, Kyu-Hyung Ryu

**Affiliations:** 1 Department of Internal Medicine, Konkuk University School of Medicine, Konkuk, South Korea; 2 Department of Cardiology, Korea Guro University Hospital, Seoul, South Korea; 3 Heart Center, Gachon University of Medicine and Science, Gil Hospital, Incheon, South Korea; 4 Department of Internal Medicine, College of Medicine, Hallym University, Seoul, South Korea; 5 Division of Cardiology, Department of Internal Medicine, Hallym University Medical Center, 94-200 Yeongdeungpo-Dong, Yeongdeungpo-Gu, Seoul, 150-719, South Korea

**Keywords:** Sudden cardiac death, Early repolarization, ST segment

## Abstract

**Background:**

Risk stratification of the early repolarization pattern (ERP) is needed to identify malignant early repolarization. J-point elevation with a horizontal ST segment was recently suggested as a malignant feature of the ERP. In this study, the prevalence of the ERP with a horizontal ST segment was examined among survivors of sudden cardiac arrest (SCA) without structural heart disease to evaluate the value of ST-segment morphology in risk stratification of the ERP.

**Methods:**

We reviewed the data of 83 survivors of SCA who were admitted from August 2005 to August 2010. Among them, 25 subjects without structural heart disease were included. The control group comprised 60 healthy subjects who visited our health promotion center; all control subjects were matched for age, sex, and underlying disease (diabetes mellitus, hypertension). Early repolarization was defined as an elevation of the J point of at least 0.1 mV above the baseline in at least two continuous inferior or lateral leads that manifested as QRS slurring or notching. An ST-segment pattern of <0.1 mV within 100 ms after the J point was defined as a horizontal ST segment.

**Results:**

The SCA group included 17 men (64%) with a mean age of 49.7 ± 14.5 years. The corrected QTc was not significantly different between the SCA and control groups (432.7 ± 37.96 vs. 420.4 ± 26.3, respectively; p = 0.089). The prevalence of ERP was not statistically different between the SCA and control groups (5/25, 20% vs. 4/60, 6.7%, respectively; p = 0.116). The prevalence of early repolarization with a horizontal ST segment was more frequent in the SCA than in the control group (20% vs. 3.3%, respectively; p = 0.021). Four SCA subjects (16%) and one control subject (1.7%) had a J-point elevation of >2 mm (p = 0.025). Four SCA subjects (16%) and one (1.7%) control subject had an ERP in the inferior lead (p = 0.025).

**Conclusion:**

The prevalence of ERP with a horizontal ST segment was higher in patients with aborted SCA than in matched controls. This result suggests that ST morphology has value in the recognition of malignant early repolarization.

## Background

The early repolarization pattern (ERP) has been recognized as a benign variant for the last six decades. Recent case control studies have, however, showed an association between idiopathic ventricular fibrillation and early repolarization [1-3]. In addition, population-based studies have reported that early repolarization is associated with an increased risk of cardiac death in the Western and Asian general populations [4-6]. These results have called attention to risk stratification of ERP to identify malignant ERP [7]. Suggested malignant features thus far are a history of familial sudden death [1], J-wave amplitude of >0.2 mV [1,3], and J-wave distribution of the inferior lead [3]. However, additional studies comparing malignant and asymptomatic ERP are needed to clarify the characteristics of malignant ERP [8]. Recent studies reported that early repolarization with ST elevation has a good prognosis [9,10], but an ERP with a horizontal ST segment is associated with idiopathic ventricular fibrillation [11]. In this study, the prevalence of the suggested malignant features of ERP, including the newly reported ERP with a horizontal ST segment, was examined among survivors of sudden cardiac arrest (SCA) without structural disease to determine whether these features are associated with a history of SCA.

## Methods

### Study population

We reviewed the data of 83 survivors of SCA who were admitted to our center from August 2005 to August 2010. We enrolled patients with a normal coronary artery on coronary angiography and no identifiable structural disease on follow-up echocardiography. Patients were excluded if a reversible cause of SCA was diagnosed (such as electrolyte imbalance or drug poisoning). Patients with Brugada syndrome, defined as right bundle branch block and ST-segment elevation of >0.2 mV in more than two precordial V1–3 leads, were excluded. We also excluded patients with a history of structural heart disease, obstructive pulmonary disease, and end-stage renal disease. A total of 25 subjects were identified as having survived SCA without structural heart disease. Electrocardiograms (ECGs) taken at the first office visit after discharge or the last ECG were reviewed. In two cases, ECGs that were taken before the cardiac arrest were available. This study was approved by the ethics review board of Konkuk University Hospital (KUH 1010063).

### Control group

The control group comprised 60 healthy subjects who visited the health promotion center of Konkuk University Hospital, Seoul, Korea from 1 December 2010 to 20 December 2010. This population was matched for age, sex, and underlying disease (hypertension and diabetes mellitus).

### Measurement and data collection

**Figure 1:**
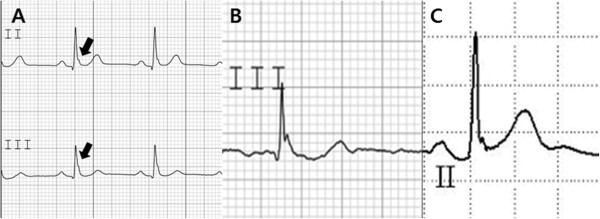
**The definition of early repolarization patterns.** (**A**) Early repolarization: defined as a QRS-ST junction elevation of at least 1 mm (0.1 mV) above the baseline in at least two continuous inferior or lateral leads that manifests as QRS slurring or notching (thick arrow). (**B**) A horizontal ST segment was defined as a J-point elevation of <0.1 mV within 100 ms after the J point (46-yr-old man in SCA group). (**C**) An ascending ST segment was defined as a J-point elevation of >0.1 mV within 100 ms after the J point (44-yr-old man in control group).

ECGs were evaluated in random order by two physicians who were blinded to the subject grouping. The presence of ERP was checked. Early repolarization was defined as a QRS-ST junction elevation of at least 1 mm (0.1 mV) above the baseline in at least two continuous inferior or lateral leads that manifested as QRS slurring or notching (Figure 1A). The amplitude of the J-point elevation was measured, and a J-point elevation of >0.2 mV was defined as a large-amplitude J-point elevation.

A horizontal ST segment was defined as a <0.1-mV elevation of the ST segment within 100 ms (Figure 1B). An ST-segment pattern elevation of >0.1 mV within 100 ms after the J point was defined as an ascending ST segment (Figure 1C). Serum LDL and total cholesterol levels and corrected QT intervals were also determined. The corrected QTc interval was calculated by Bazett’s formula.

### Statistical analysis

Continuous variables are displayed as mean ± SD, and categorical variables are presented as number and percentage. Continuous variables between the SCA and control groups were compared using Student’s *t* test. Categorical variables were compared with the two-tailed Fisher’s exact test. A p value of <0.05 was considered statistically significant. Statistical analysis was performed with PASW statistical software (version 17).

## Results

The SCA group included 16 men and 9 women with a mean age of 49.6 ± 12.7 years. The control group included 60 subjects who were well matched for age (52.2 ± 16 years), sex (37 men and 23 women), and prevalence of hypertension (12% vs. 25%, respectively; p = 0.249) and diabetes mellitus (12% vs. 16%, respectively; p = 0.747) (Table 1).

**Table 1:** Clinical characteristics of sudden cardiac arrest group and control group

	**Sudden cardiac arrest**	**Control**	**p value**
Subjects, n	25	60	
Male, n (%)	16 (64)	37 (61.6)	p = 1.0
Hypertension, n (%)	3 (12)	15 (25)	p = 0.249
DM, n (%)	3 (12)	10 (16)	p = 0.747
Age, years	49.6 ±12.7	52.2 ± 16	p = 0.274
QTc, ms	432.7 ± 37.96	420.4 ± 26.3	p = 0.089
LDL, mg/dl	87 ± 37.6	112 ± 27.8	p = 0.02

The corrected QTc was not significantly different between the SCA and control groups (432.7 ± 37.96 vs. 420.4 ± 26.3, respectively; p > 0.05), but the serum LDL level was significantly higher in the control group (83 ± 37.6 vs. 112 ± 27.8, p = 0.02). In the analysis of the presenting ECG at the time of admission, 10 (40%) subjects had ventricular arrhythmia, 1 (4%) had pulseless electrical activity, and 14 (56%) had asystole. J-point elevation was present in five survivors (20%) of SCA and four (6.7%) control subjects (Table 2). However, the prevalence of J-point elevation was not statistically different (p = 0.116). The prevalence of early repolarization with a horizontal ST segment was more frequent in the SCA group than in the control group (20% vs. 3.3%, respectively; p = 0.021). J-point elevation of >2 mm was more common in the SCA group than in the control group (16% vs. 1.7%, respectively; p = 0.025). More aborted SCA subjects had an ERP in the inferior lead compared with the control group (16% vs. 1.7%, respectively; p = 0.025).

**Table 2:** Incidence of early repolarization and characteristics among study group and control group

	**Sudden cardiac arrest**	**Control**	**p value**
Early repolarization (inf+lat) n, (%)	5 (20)	4 (6.7)	p = 0.116
J-point elevation of >2 mV, n (%)	4 (16)	1 (1.7)	p = 0.025
ER with horizontal ST	5 (20)	2 (3.3)	p = 0.021
ER with ascending ST	0 (0)	2 (3.3)	p > 0.05
Distribution of ER			
Inferior lead, n (%)	4 (16)	1 (1.7)	p = 0.025
Lateral lead, n (%)	1 (4)	3 (5)	p = 1.0

## Discussion

Recent studies have raised concerns about ERP-related SCA. However, little data on risk stratification of ERP are available. In 1961, Wasserburger et al. defined early repolarization as an ST-segment elevation at the J junction of the QRS complex accompanied by downward concavity of the ST segment and suggested that it is a normal variant [12]. Subsequent long-term studies failed to find any adverse consequences [13,14]. In 2000, Gussak et al. suggested that an ERP may cause malignant arrhythmias by showing that the ERP reflecting dispersion of repolarization in arterially perfused canine left ventricle wedge preparations facilitates ventricular arrhythmia by phase-2 re-entry [15]. Case control studies have shown that the prevalence of ERP was statically higher in the idiopathic ventricular fibrillation group than in the controls. In 2008, Haissaguerre et al. and Nam showed a significant association between ERP and ventricular fibrillation in their case control studies [1,2]. Furthermore, subsequent studies based on general population cohorts suggested that an ERP is associated with sudden cardiac death in Western and Asian populations [4-6].

However, it is inappropriate to compare previous studies with recent studies suggesting ERP is associated with SCA because the definition of ERP in recent studies differs from that of Wasserburger [16]. The classical definition of ERP focused on the downward concavity of the ST segment in the lateral leads because of its critical association with myocardial infarction and pericarditis [10]. On the other hand, a new definition suggested by Haissaguerre et al. only requires a slurred or notched J-point elevation of >0.1 mV in two contiguous inferior or lateral leads; ST-segment elevation is not necessary. In a recent study, Tikkanen et al. subgrouped the ERP into an ascending ST segment and horizontal ST segment by ST-segment morphology [9]. The definitions of ascending ST segment and horizontal ST segment in the present study originated from their work. These definitions represent ERP subgroupings as one compatible with the old description and another that was not originally considered to be early repolarization [16]. They showed that an ERP with a horizontal/descending ST segment, but not an ERP with an ascending ST segment, was associated with an increased risk of sudden cardiac death in the general population [9]. Uberoi et al. also found no significant association between an ERP with an ascending ST segment and cardiac mortality in their outpatient-based retrospective population study [10]. Another recent study by Rosso et al. reported that J-point elevation with a horizontal ST segment has a three-fold higher odds ratio for idiopathic ventricular fibrillation compared with J-point elevation only (OR, 13.8 and 95 % CI, 5.1–37.2 vs. OR, 4 and 95% CI, 2.0–7.9, respectively) and concluded that the combination of J-point elevation with a horizontal ST segment improves the ability to distinguish malignant forms of ERP [11]. Interestingly, the ERP itself, which was suggested to be a predictor of sudden cardiac death, was not associated with SCA in this study; however, an ERP with a horizontal ST segment was associated with SCA. This may have resulted not only from the small number of subjects in this study, but also the low relative risk of early repolarization per se [5]. The results of this study support the conclusions of the studies by Tikkanene et al. and Rosso et al. Other suggested malignant features (J-point elevation of >0.2 mV and ERP location in the inferior wall) were associated with SCA, and this result is also consistent with those of previous studies [3,5]. To the best of our knowledge, this study is the first case control study to examine the prevalence of malignant ERP features in patients with SCA. The prevalence of the ERP in the control group was 6.7%. This result is comparable with that in a study by Tikkanen et al., who showed that the prevalence of ERP was 5.8% among 10,864 middle-aged North European subjects [5]. According to the study by Uberoi et al., the prevalence of the ERP with an ascending ST segment among 29,181 Americans was 2.3%, and it was 3.3% in the control group of this study [10].

Explanation for the favorable outcomes of an ERP with an elevated ST segment is not sufficient. In the study by Tikkanen et al., the subjects in the ERP group with an ascending/upsloping ST segment were younger and had a lower blood pressure, lower heart rate, shorter QTc duration, and higher prevalence of left ventricular hypertrophy on ECG. These results suggest that an ERP with an ascending ST segment might occur secondary to ECG changes in athletes [9]. In addition, Bastiaenen et al. showed that early repolarization with a horizontal ST segment may be associated with abnormal depolarization based on the finding that subjects with early repolarization with a horizontal ST segment had a longer QRS duration [16]. However, the evidence for abnormal depolarization in early repolarization syndrome is still controversial [17], and the association between early repolarization with a horizontal ST segment and late depolarization has not been well studied.

### Study limitations

First, the number of subjects was small. Further large and prospective population-based studies are needed to confirm the risk of early repolarization with a horizontal ST segment. Second, electrophysiology tests were not performed in this study. Recent studies demonstrated that the prevalence of abnormal late potentials, which are usually associated with depolarization abnormality, is not low, suggesting that the ERP is associated with depolarization abnormality [18,19]. Thus, further studies involving large numbers of patients with SCA and associated ERP are needed to understand the underlying pathophysiology of malignant ERP. Third, the provocation to induce coronary spasm could not be performed in the aborted SCA group because of the inversed risk-benefit balance. Obtaining a detailed history during the post-cardiac arrest follow-up would limit the possibility of coronary spasm.

## Conclusion

In this study, we demonstrated that an ERP with a horizontal ST segment was more prevalent in the SCA group than in the matched controls. This result confirms those of prior general population-based studies and case control studies and suggests that all ERPs might not be generated in the same way because ST-segment morphology seems to have important value in the risk stratification of early repolarization.

## Competing interests

The authors declare that they have no competing interests.

## Authors’ contributions

Sung Hea Kim and Do Young Kim participated in data analysis and drafted the initial manuscript. Hyun-Joong Kim and Soon Yong Suh participated in the sequence alignment and critical revision. Sang Man Jung and Seong Woo Han participated in the design of the study and performed the statistical analysis. Kyu-Hyung Ryu conceived the study, participated in its design and coordination, and helped to draft the manuscript. All authors made substantial contributions to the acquisition of data. All authors read and approved the final manuscript.

## Pre-publication history

The pre-publication history for this paper can be accessed here:

http://www.biomedcentral.com/1471-2261/12/122/prepub
